# FTO polymorphism (RS9939609) and BED in patients with morbid obesity for evaluation towards bariatric surgery

**DOI:** 10.1186/1758-5996-7-S1-A146

**Published:** 2015-11-11

**Authors:** Jaqueline Driemeyer C Horvath, Mariana Laitano Dias de Castro, Natalia Luiza Kops, Rogério Friedman

**Affiliations:** 1 UFRGS/UCS, Porto Alegre, Brazil

## Background

Obesity is a major health problem in the twenty-first century. Recent research shows that, among people with severe obesity, there is a sub-population that does not respond to behavioral treatment for weight loss. This group presents a type of eating disorder known as Binge Eating Disorder (BED). Recent studies indicate a link between obesity in adults and the rs9939609 polymorphism of the FTO gene. A frequent association of obesity and BED has also been found. Variations in the gene, including the rs9939609 polymorphism, have been associated with obesity and diabetes.

## Objective

To identify whether there is any associaton between the rs9939609 polymorphism of FTO, and the presence of BED in severely obese patients.

## Materials and methods

Patients referred to the Endocrine clinic of Hospital de Clinicas de Porto Alegre, with morbid obesity for evaluation towards bariatric surgery, according to the Brazilian guidelines, between January 2010 and December 2013, were studied. The patients answered a structured questionnaire (Binge Eating Scale) for diagnosis of BED. A blood sample was drawn for analysis of the candidate gene polymorphism, and biochemical parameters (fasting plasma glucose, A1c, lipids).

## Results

We studied, sequentially, 160 patients, aged (mean±SD) 44.5±11.5 yrs., 78.8% female. 56.7% without BED, 22.3% with severe compulsion and 21% with moderate compulsion. The frequencies of the rs9939609 polymorphism were 21.9% (TT), 41.9% (AT), and 36.3% (AA). BMI was 47.8±7.3 kg/m^2^, weight was 126.8±24.1 kg, waist circumference was 135.2±15.3 cm, fasting glucose 122.3±38 mg/dL. Waist circumference and fasting plasma glucose were significantly different between the three genotypes (ANOVA, p=0.014, and p=0.030, respectively). Waist circumference (140.3±15.9 cm) was higher in the homozygous AA group. Fasting plasma glucose was lower in the AA group (110.8±22.7 mg/dL). Nevertheless, the frequency of BED was not different between the groups, and none of the other variables was different in any of the 3 groups.

## Conclusion

In severely obese human subjects, the rs9939609 polymorphism of FTO is not associated with BED.

